# Risk factors for re-admittance due to allergic food reactions in a cohort of patients from pediatric emergency departments in Stockholm

**DOI:** 10.1186/2045-7022-3-S3-P106

**Published:** 2013-07-25

**Authors:** M Vetander, DH Ly, N Håkansson, M Wickman, A Bergström

**Affiliations:** 1 Sachs’ Children’s Hospital, Department of Paediatrics, Södersjukhuset, Stockholm, Sweden; 2 Karolinska Institutet, Department of Clinical Science and Education, Södersjukhuset, Stockholm, Sweden; 3 Institute of Environmental Medicine, Karolinska Institutet, Stockholm, Sweden; 4 Institute of Environmental Medicine, Karolinska Institutet, Stockholm, Sweden

## Background

Knowledge about repeated food allergic reactions in a pediatric emergency department (ED) setting is sparse. We aimed to investigate potential risk factors for ED re-visits, and to determine the risk of future more severe reactions among children with a prior ED visit due to allergic reactions to food.

## Methods

In this cohort study the study population consisted of 358 children with ED visits at any of the three pediatric hospitals in Stockholm County due to allergic reactions to foods during 2007 (hereafter called the index-reaction). Re-visits due to allergic reactions to foods during the follow-up period (1 January 2007 - 30 June 2010) were identified using medical records. Cox proportional hazard models adjusting for potential confounders were used to estimate relative risks.

## Results

80 children had a total of 116 ED re-visits over a period of 873 patient-years, yielding an incidence rate of ED re-visits of 9/100 patient-years. Known food allergy before the index ED visit in 2007 were identified as a risk-factor for ED re-visits with a more pronounced tendency towards risk among children with two or more food-allergies, compared to children with one food allergy (relative risk (RR) = 2.79, 95% confidence interval (CI): 1.56-5.00, RR = 1.87, 95% CI: 1.01-3.46, respectively). Prescription of adrenaline auto injector before the index-reaction also emerged as a risk-factor for ED re-visits (RR = 2.02, 95% CI 1.17-3.49). We found no statistically significant associations with other potential risk-factors. When comparing the severity of the reaction at the ED re-visit with the index-reaction, 21% had more severe reactions, 38% less severe reaction and 41% reactions of comparable severity. However, among 29 (36%) of the children, treatment with adrenaline hampered the classification of change in severity. Among children with anaphylaxis at the ED re-visit prescribed with adrenaline before the visit (n=17), 13 (76%) had not used the device.

## Conclusion

Besides known food allergy and prescription of adrenaline before the index-reaction we found no statistically significant risk factors for ED re-visits in our study. The severity of the reaction at the ED re-visit could not be predicted by the severity of the index-reaction and classification of change in severity was often hampered by treatment with adrenaline. A major concern was that the vast majority of children with anaphylaxis prescribed with adrenaline failed to use their device.

## Disclosure of interest

None declared.

